# A Comparative Review of Key Isothiocyanates and Their Health Benefits

**DOI:** 10.3390/nu16060757

**Published:** 2024-03-07

**Authors:** Julia B. Olayanju, Dragica Bozic, Uma Naidoo, Omowunmi A. Sadik

**Affiliations:** 1 Department of Biology, Saint Elizabeth University, Morristown, NJ 07960, USA; 2 FoodNiche Institute, Mount Olive, NJ 07828, USA; 3 Department of Toxicology, School of Pharmacy, University of Belgrade, 11221 Belgrad, Serbia; 4 Harvard Medical School, Boston, MA 02115, USA; 5 Massachusetts General Hospital, Boston, MA 02114, USA; 6 Department of Chemistry, New Jersey Institute of Technology, Newark, NJ 07102, USA

**Keywords:** isothiocyanate, chemoprevention, anticarcinogenic, antitumor

## Abstract

Isothiocyanates are biologically active products resulting from the hydrolysis of glucosinolates predominantly present in cruciferous vegetables belonging to the *Brassicaceae* family. Numerous studies have demonstrated the diverse bioactivities of various isothiocyanates, encompassing anticarcinogenic, anti-inflammatory, and antioxidative properties. Nature harbors distinct isothiocyanate precursors, glucosinolates such as glucoraphanin and gluconastrin, each characterized by unique structures, physical properties, and pharmacological potentials. This comprehensive review aims to consolidate the current understanding of *Moringa* isothiocyanates, mainly 4-[(α-L-rhamnosyloxy) benzyl] isothiocyanate), comparing this compound with other well-studied isothiocyanates such as sulforaphane and phenyl ethyl isothiocyanates. The focus is directed toward elucidating differences and similarities in the efficacy of these compounds as agents with anticancer, anti-inflammatory, and antioxidative properties.

## 1. Introduction

Isothiocyanates, a class of compounds predominantly found in cruciferous plants such as broccoli, cauliflower, kale, cabbage, watercress, and others, impart these vegetables with their characteristic spicy and bitter taste. Epidemiological studies dating back to the early 1990s have indicated that an increased intake of these vegetables can yield beneficial health effects and has the potential to mitigate the risk of developing certain diseases. These hypotheses were subsequently corroborated by clinical studies [1,2]. Isothiocyanates emerge as biologically active products resulting from the hydrolysis of glucosinolates, secondary metabolites found in plants from the *Brassicaceae* family. The conversion of glucosinolates into bioactive forms occurs through the process of hydrolysis, catalyzed either by the endogenous enzyme myrosinase (β-thioglucosidase) or human gastrointestinal microbiota [3,4,5]. The activation of glucosinolates into their active metabolites takes place when plants are exposed to various stressors such as infections or mechanical damage. Consequently, the formation of isothiocyanates serves as a defensive mechanism protecting the plant from injury [6,7]. This intriguing observation prompted further investigations into isothiocyanates, aiming to unravel the underlying mechanisms facilitating plant survival in diverse conditions. Studies have revealed that isothiocyanates encompass a plethora of health benefits, including antidiabetic, anticancer, analgesic, and cardioprotective effects, the potential to treat neurological disorders, and regulation of thyroid gland function [8,9,10]. Thus, the purpose of this review is to consolidate current understanding about well-studied isothiocyanates such as sulforaphane and phenyl ethyl isothiocyanate, and to compare them with less investigated moringa isothiocyanates (MIC). The focus is directed toward elucidating differences and similarities in the anticancer, anti-inflammatory, and antioxidative efficacy of these compounds. 

**Figure 1:**
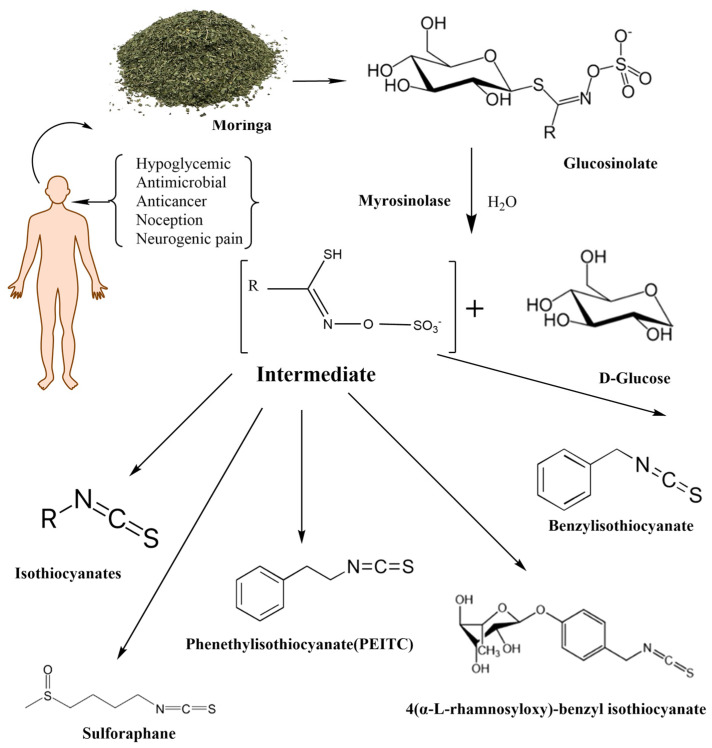
The enzymatic reactions of glycosylate with myrosinase and their degradation products.

Mentioned representatives of this compound class, phenethyl isothiocyanate (PEITC or chemically 2-isothiocyanato ethylbenzene), sulforaphane (SFN or 1-isothiocyanato-4-methylsulfinylbutane), and MIC (4-[(α-L-rhamnosyloxy)benzyl] isothiocyanate), have been investigated (Figure 1). As illustrated in Table 1, these bioactive compounds exhibit variations in structure and physical properties.

### 1.1. Phenethyl Isothiocyanate

Watercress (*Nasturtium officinale*) stands out as an easily accessible garden vegetable, abundant in gluconasturtiin—an aromatic glucosin olate featuring an ethyl chain linked to benzene in its radical. The hydrolysis of gluconasturtiin by myrosinase yields PEITC, an isothiocyanate with a phenylethyl radical attached to a nitrogen atom [11]. Upon activation, PEITC showcases neuro- and cardioprotective effects, along with antitumor and antimutagenic properties, contributing significantly to the prevention of carcinogenesis and other chronic degenerative diseases [12,13,14,15]. The pivotal mechanism driving PEITC bioactivity involves the activation of molecular pathways regulated by the transcription factors nuclear factor erythroid 2-related factor 2 (Nrf2) and heat shock factor 1 (HSF1). The sulfhydryl group of PEITC binds to cysteine residues in its protein targets, initiating the molecular process [16,17]. This review will delve further into the intricate interplay between PEITC and Nrf2, providing a comprehensive exploration of this critical mechanism.

### 1.2. Sulforaphane

Sulforaphane emerges as a metabolic product originating from the stable phytochemical glucoraphanin (GPN), a glucosinolate derived from dihomomethionine. The conversion of GPN to SFN transpires during the cutting or chewing of broccoli sprouts, a process that exposes GPN to the catalytic action of the myrosinase enzyme [18]. Similar to other isothiocyanates, SFN has demonstrated a myriad of health benefits, encompassing anti-inflammatory and antioxidant effects, inhibition of tumor cell growth, antidiabetic properties, cardioprotective effects, and other advantageous health impacts [19,20,21]. The isothiocyanate functional group within the SFN molecule emerges as the crucial pharmacophore, dictating its bioactivity [22]. SFN has garnered increased popularity in recent years, primarily due to its recognized anticancer potential. Research reports suggest that SFN induces apoptosis in tumor cells through both intrinsic and extrinsic apoptotic pathways [23]. Furthermore, SFN inhibits cancer initiation by modulating metabolic enzymes, leading to the reduction of carcinogen-activating phase I enzymes (e.g., decreasing CYP1A1 (cytochrome P450 family 1 subfamily A member 1) and CYP3A4 (cytochrome P450 family 3 subfamily A member 4) activity) and activation of carcinogen-detoxifying phase II enzymes [22,24]. 

### 1.3. Moringa Isothiocyanates

*Moringa* isothiocyanates are the least studied of the isothiocyanates. Similar to other isothiocyanates, they are found in a precursor form in *Moringa oleifera*, a tropical plant abundant in glucosinolates [25]. *Moringa* glucosinolates exhibit a unique structure with an additional sugar group. Four bioactive and relatively stable isothiocyanates are formed from *Moringa* glucosinolates, with 4-[(α-L-rhamnosyloxy) benzyl] isothiocyanate and 4-[(4′-O-acetyl-α-L-rhamnosyloxy)benzyl] isothiocyanate comprising over 95% of the total MIC, while others are present in smaller amounts [26]. Moringin (MG), as the most abundant MIC, results from myrosinase-catalyzed hydrolysis of glucomoringin (GMG). Alongside PEITC and SFN, MG has attracted attention across various research domains, including cancer, antimicrobial applications, neurodegenerative diseases, and more, owing to its anti-inflammatory and antioxidative effects (Figure 1) [27].

For instance, transcriptomic analysis revealed MG’s capacity to suppress inflammation and oxidative stress by reducing the expression of inflammatory cytokines, such as TNF-α (tumor necrosis factor α), IFN-α (interferon α), IL-1β (interleukin 1β), and IL-6 (interleukin 6), while simultaneously increasing Nrf2 gene expression and its nuclear accumulation. This resulted in a decrease in NF-κB translocation and its binding to promoter sites on responsive genes, coupled with a reduction in reactive oxygen species production. MG also demonstrated antioxidative characteristics, overcoming colchicine-induced oxidative stress in an Alzheimer’s disease rat model and improving the rats’ memory [12,13].

## 2. Isothiocyanate Metabolism

Upon hydrolyzation of glucosinolates in the gastrointestinal tract, isothiocyanates seamlessly enter the bloodstream and form associations with plasma proteins. This binding facilitates their traversal across the plasma membrane of cells, allowing entry into the cellular milieu. Once inside the cells, isothiocyanates engage in a reaction with glutathione, catalyzed by the enzyme glutathione S-transferase (GST). Subsequently, this conjugate is transported to the extracellular medium, where γ-glutamyl transferase and dipeptidase enzymatically dismantle its γ-glutamyl and glycyl components. The resulting metabolite then undergoes transportation to the liver, where it enters the mercapturic acid pathway. Within this pathway, *N*-acetyl transferases acetylate the metabolite, leading to the formation of the *N*-α-acetyl derivative or mercapturic acid. Finally, the formed derivatives are conveyed to the kidneys and actively expelled into the urine, completing the process of elimination from the body [28,29].

### 2.1. Metabolism of Phenethyl Isothiocyanate

PEITC demonstrates rapid absorption and notable bioavailability. The primary metabolic pathway for PEITC involves glutathione conjugation, leading to excretion in both urine and bile in the form of mercapturate. In the liver, the enzyme GST facilitates the addition of a glutathione (GSH) tail to PEITC, forming PEITC-GSH. Further metabolism occurs as γ-glutamyl transferase and dipeptidase act on the γ-glutamyl and glycyl moieties of PEITC-SG, respectively. The resulting cysteine conjugate undergoes *N*-acetylation of its cysteine radical through the action of *N*-acetyltransferase 2 (NAT2) and is eventually excreted as mercapturic acid in the urine [30,31].

### 2.2. Metabolism of Sulforaphane

Upon entering the body, SFN undergoes metabolism through the mercapturic acid pathway. The interaction involves the reactive electrophilic carbon from the isothiocyanate functional group (−N=C-S) and GSH, catalyzed by GST. The resulting SFN-GSH complex undergoes further transformations by enzymes such as γ-glutamyltranspeptidase, cysteinylglycinase, and *N*-acetyltransferase. The main metabolite formed is SFN-*N*-acetylcysteine. SFN exhibits high bioavailability, undergoes rapid metabolism, and is excreted from the body through urine. Once inside the cells, it accumulates [4,6].

### 2.3. Metabolism of Moringa Isothiocyanates

Although no data are available about the metabolic transformation of *Moringa* isothiocyanates, it is anticipated that they enter the metabolic pathway of mercapturic acid similarly to other isothiocyanates. As reported for PEITC and SFN, *Moringa* isothiocyanates undergo consecutive reactions involving GST, γ-glutamyl transferase, and dipeptidase. Eventually, NAT2 converts them into mercapturic acid, which is then excreted via the kidneys [4,6,29,30,31]. However, a recent study conducted in rats detected MIC in the serum without chemical or enzymatic degradation, indicating that MIC is protected from typical isothiocyanate metabolism and other metabolic modifications because of its unique glycosidic motif [32], but further confirmations are needed. Moreover, it was reported that the structural difference from other isothiocyanates does not impact MIC efficacy but rather contributes to their stability. As stated, purified MIC is a white crystalline powder stable at 25 °C, while SFN and PEITC are volatile, viscous liquids [33].

## 3. Chemical Structures of the Three Isothiocyanates

Functional similarity among isothiocyanates arises from the isothiocyanate moiety (−N=C=S), a functional group formed by substituting the oxygen in the isocyanate group with sulfur. This group forms hydroxyl bonds with sulfhydryl, hydroxy, and amine groups on targeted proteins. The newly formed bonds are reversible, meaning that the activity of isothiocyanates ends after the hydrolysis of proteins [34].

*Moringa* isothiocyanates exhibit solid and relatively stable properties at room temperature, as a result of the additional rhamnose sugar group attached to the isothiocyanate moiety. On the other hand, PEITC and SFN are volatile molecules available in liquid form [26,35]. Isothiocyanates are liposoluble molecules with the ability to cross the blood–brain barrier, giving them neuroprotective activity against conditions such as Alzheimer’s disease, Parkinson’s disease, and multiple sclerosis [36,37].

These plant-derived isothiocyanates are well-known for their antioxidative and anti-inflammatory activity, which may be responsible for various beneficial health effects reported in the literature.

## 4. Nrf2 Activation and Regulation of Oxidative Stress

The activation of the Nrf2 transcription factor and consequently the inhibition of nuclear factor kappa B (NF-κB) signaling resulting in protection from oxidative stress is one of the most investigated mechanisms for PEITC and SFN. Upon translocation into the nucleus, Nrf2 activates the antioxidant response element (ARE) and stimulates the transcription of antioxidant and cytoprotective enzymes [37,38]. The interaction between Nrf2 and ARE leads to the upregulation of heme oxygenase 1 (HO-1), an enzyme that catalyzes the degradation of heme into CO, bilirubin, and free iron. HO-1 inhibits the production of pro-inflammatory cytokines such as IL-6 and TNF-α while stimulating the secretion of anti-inflammatory interleukins, mainly interleukin 10 (IL-10). Moreover, the HO-1-mediated release of CO leads to inhibition of the NF-ĸB signaling pathway and thus, further decreases the expression of inflammatory molecules [39]. Nrf2 mediates the transcription of other detoxification and antioxidant genes, such as NAD(P)H-quinone oxidoreductase 1 (NQO1), glutathione S-transferases (GSTs), aldo-keto reductases (AKRs), aldose reductase (AR), γ-glutamyl peptidase (GGT), carboxylesterase (CES), UDP-glucuronosyltransferases (UGTs), thioredoxin (TXN), and glutamate-cysteine ligase catalytic/modifier subunits (GCLC/GCLM) [17], indicating the existence of a complex interplay between inflammation and oxidative stress where Nrf2 plays a central role.

All isothiocyanates react with Nrf2 in the same way. The electrophilic center of isothiocyanates interacts with thiol (-SH) groups of cysteine residues in Kelch-like ECH-associated protein 1 (Keap1), forming a stable bond between sulfur and carbon in a process called Michael addition. This leads to the release of Nrf2 from its complex with Keap1, stabilization of Nrf2 protein, and its translocation to the nucleus [40,41,42]. It is claimed that SFN, PEITC, and MIC have a high affinity for Keap1 [42]; however, their dissociation constant (Kd) has not been determined yet. Thus, the isothiocyanates–Keap1 interaction might be dependent on other factors, such as the dose of isothiocyanate used in a reaction, the time of exposure, the type of cells exposed to isothiocyanates, or other factors.

### 4.1. Role of Phenethyl Isothiocyanate in Nrf2 Activation and Activities

Phenethyl isothiocyanate has been shown to activate Nrf2 in both in vitro and in vivo studies. Ernst et al. (2011) explained that PEITC increases the phosphorylation of extracellular signal-regulated (ERK1/2) kinases 1/2, which are responsible for Nrf2 phosphorylation and translocation to the nucleus [40]. In a study by Eisa et al. (2021), the nephroprotective role of PEITC in diabetic mice was found to be Nrf2-dependent, stimulating the expression of downstream targets such as HO-1 and γ-GCS (gamma glutamate-cysteine), proteins that counteract oxidative stress [43].

However, other anti-inflammatory mechanisms of PEITC have also been proposed. In obese mice treated with PEITC, higher levels of NF-κB, lectin-like oxidized low-density lipoprotein receptor 1 (LOX-1), and cyclooxygenase-2 (COX-2) were observed compared to the control group. PEITC supplementation was able to decrease the expression levels of NF-κB, LOX-1, and COX-2, ameliorating obesity-induced inflammation via mTOR/PPARγ/AMPK signaling (mammalian target of rapamycin/peroxisome proliferator-activated receptor gamma/adenosine-monophosphate activated-protein kinase) [40,44]. PEITC has also shown beneficial effects against bacterial and viral infections by suppressing Toll-like receptor signaling. This leads to the downregulation of NF-κB and interferon regulatory factor 3 (IRF3), ultimately inhibiting interferon β (IFNβ) and interferon-inducible protein-10 (IP-10), which are genes involved in infection-triggered inflammation [45].

### 4.2. Role of Sulforaphane in Nrf2 Activation and Activities

Sulforaphane has been associated with the modulation of Nrf2/Keap and NF-κB, contributing to protection against cardiovascular-related inflammation, atherosclerosis, hypertension, diabetes mellitus, cardiomyopathy, and heart failure [12].

For instance, Pan et al. (2023) demonstrated that SFN can reduce vascular remodeling in hypoxic pulmonary hypertension by reactivating Nrf2, decreasing the activity of effector T cells, and suppressing the production of inflammatory molecules such as TNF-α and IL-6. Additionally, SFN enhanced antioxidative defense mechanisms by improving superoxide dismutase (SOD) activity and increasing total glutathione levels [46]. The anti-inflammatory properties of SFN were also observed in an animal model of diabetic cardiomyopathy [47,48]. Mice with diabetes treated with SFN for 4 months showed lower risks of developing cardiac dysfunction, oxidative damage, inflammation, fibrosis, and hypertrophy compared to a control group [47,48]. Similarly, Sun and colleagues reported that SFN-mediated activation of Nrf2 signaling can prevent cardiomyopathy in mice with streptozotocin-induced hyperglycemia in an AMPKα2-dependent manner. AMPKα2 is an immune-suppressive protein that downregulates the activation of NF-κB signaling [49].

### 4.3. Role of Moringa Isothiocyanates in Nrf2 Activation and Activities

Most studies on *Moringa* isothiocyanates focus on their anti-inflammatory activities, demonstrating beneficial roles in conditions such as diabetic nephropathy, microbial infections, and neuroprotection [10,50,51]. The anticipated immune-suppressive mechanism of MIC was found to be Nrf2-dependent. For instance, exposing human renal proximal tubule HK-2 cells to increasing doses of MG (1.25–5 μM) resulted in the upregulation of Nrf2 gene expression, particularly NQO1, HO-1, and GCLC, while inhibiting transforming growth factor beta 1 (TGFβ1). Consequently, MG exhibited a dual action by suppressing inflammation and reducing oxidative stress in the treated cells through the common isothiocyanate mechanism of action [50]. Likewise, MG demonstrated the ability to prevent renal injury in diabetes-bearing mice by repressing ROS and malondialdehyde (MDA) levels, while enhancing the production and secretion of defensive proteins like GSH, SOD, and catalase (CAT). At the molecular level, this was associated with the induction of Nrf2 signaling and the inhibition of NF-κB activity [52]. Additionally, the neuroprotective effects of MG were observed in various neurodegenerative disorders, including Parkinson’s Disease, Alzheimer’s Disease, Huntington’s Disease, and Amyotrophic Lateral Sclerosis, where once again, Nrf2 activation played a central role [10]. The documented beneficial effects of isothiocyanates against various cancers highlight the proposed induction of both intrinsic and extrinsic apoptotic pathways as the most crucial underlying mechanism.

## 5. Antitumor Activities of Isothiocyanates

### 5.1. Antitumor Activities of Phenethyl Isothiocyanate

Phenethyl isothiocyanate is one of the most studied isothiocyanates with well-established antitumor characteristics. In vitro studies have demonstrated that PEITC induces oxidative damage in cervical cancer cells, triggering caspase-3 mediated apoptosis [53]. Similarly, colon cancer cells treated with PEITC, either alone or in combination with irinotecan, experienced higher levels of ROS and Ca^2+^ compared to the control. Authors explained that in a state of oxidative stress, cancer cells activate apoptosis as a defense mechanism, along with the Nrf2 pathway, which promotes the production of antioxidative proteins such as HO-1 and GSH [54]. Consequently, PEITC and/or irinotecan were able to indirectly reduce tumor growth and progression by shifting the balance of oxidation–reduction reactions to the left.

The potential of PEITC to impair the cancer cell cycle and inhibit cell viability and proliferation in four human osteosarcoma cell lines has been clearly demonstrated [55]. Multiple cell death modalities were detected, including ferroptosis, apoptosis, and autophagy, as a result of increased oxidative stress and GSH depletion. These findings were confirmed in a xenograft osteosarcoma mouse model treated with 30 mg/kg of PEITC [55].

In a mouse transgenic model of prostate cancer, a diet with 0.05% PEITC led to a reduction in tumor incidence through several mechanisms. PEITC inhibited the cell cycle in cancer cells, reduced inflammation, and disrupted cancer-related signaling [56]. Similar results were reported by Stan and colleagues in 2014, where PEITC inhibited the growth of prostatic cancer cells both in vitro and in vivo. The effect was dose-dependent, with 7 μmol/L proposed as IC50. PEITC suppressed tumor growth in vivo by stimulating G2/M phase cell cycle arrest and apoptosis. The antiapoptotic proteins B-cell lymphoma 2 (Bcl-2) and B-cell lymphoma-extra large (Bcl-XL) were downregulated in treated mice, while the expression of the proapoptotic protein Bcl-2 homologous antagonist killer (Bak) was increased [57].

Recent data indicated that PEITC can reduce the tumor’s potential to metastasize. For instance, Zhang et al. (2021) noted the ability of PEITC to reduce breast cancer metastasis via epigenetic reactivation of the tumor suppressor gene cadherin (CDH1). Reactivated CDH1 suppressed the Wnt/β-catenin pathway, which confers breast cancer stem cell properties in breast cancer cells [58]. Similar PEITC activity was observed in colorectal cancer stem cells (CSC), where it significantly reduced stem cell properties, such as clonogenicity and the expression of pluripotent factors in vitro. When PEITC-pretreated CSC were inoculated in mice, a significant reduction in tumor growth and progression was observed, in contrast to the group where mice inoculated with CSC were treated with PEITC. Thus, the authors concluded that PEITC might contribute to the prevention or delay of colorectal cancer growth by inhibiting stem cells [59].

Additionally, PEITC is often combined with other natural cytotoxic molecules or chemotherapeutics for a more pronounced antitumor effect. Results show that the combination of PEITC, indole-3-carbinol, xanthohumol, and resveratrol is more efficient in the activation of Nrf2 and NF-κB inhibition in pancreatic cancer cells than either of the substances alone [60]. Kasukabe and colleagues investigated the combination of cotylenin A and PEITC in pancreatic cancer cells resistant and non-resistant to gemcitabine and found that the combined treatment synergistically induced the generation of ROS, leading to more pronounced cancer cell death in both cell lines [61]. Finally, Li et al. (2016) demonstrated the ability of PEITC to reverse the resistance of biliary tract cancer cells to cisplatin [62].

### 5.2. Antitumor Activities of Sulforaphane

Numerous in vivo animal studies have indicated the antitumor potential of SFN. For instance, a study on rats demonstrated that SFN significantly reduces the proliferation of triple-negative breast cancer cells. Moreover, it diminished the number of stem-like cancer cells responsible for resistance to chemotherapy and radiotherapy [63]. Similarly, SFN exhibited a dose-dependent suppression of proliferation, induction of apoptosis, and reduction of lymph metastasis in female breast tumor-bearing mice [64]. The anti-tumor efficacy of SFN was observed in transgenic pancreatic cancer mice treated with 50 mg/kg of SFN for 120 days [65].

In lung tumors, SFN prevents the epithelial–mesenchymal transition, a process involved in all stages of lung cancer development and progression. This resulted in decreased invasiveness and migratory capacity of lung cancer cells via induction of MAPK/ERK signaling (mitogen-activated protein kinase/extracellular-signal-regulated kinase signaling) [66]. Li et al. (2020) identified the activation of caspase-mediated apoptosis as the key mechanism of SFN-induced tumor regression. However, they also noted that the activation of Nrf2 could stimulate autophagy, a process that helps tumors survive in a nutrition-depleted environment [67]. Lu et al. (2021) demonstrated that SFN, at a dose of 5 mg/kg b.w., induces tumor-protective autophagy, and the addition of an autophagy inhibitor increased SFN sensitivity in esophageal squamous cell carcinoma [68]. Conversely, Byun et al. showed that 5 mg/kg b.w. of SFN reduced the size of colon tumors by increasing the activity of proteins involved in the cell cycle. In vitro analysis revealed that SFN decreased cellular levels of glutathione, leading to increased ROS production in cancer cells. Elevated ROS levels further activated Nrf2 signaling and the stress-activated kinase, p38, ultimately resulting in cell cycle arrest and apoptosis [69].

Additionally, some studies demonstrated that SFN can increase cancer cell sensitivity to commonly used antitumor drugs. For example, DNA damage-induced apoptosis was more pronounced in breast cancer cells treated with a combination of cisplatin and SFN than in cells treated with only one of the mentioned drugs [70]. Similarly, combinational treatment of imatinib (IM) and SFN stimulated apoptosis of IM-resistant leukemia stem cells (LSCs). Mechanistic studies showed that combined treatment induced ROS production and subsequent activation of apoptotic molecules, namely, caspase 3, poly (ADP-ribose) polymerase (PARP), and Bcl-2-associated X protein (Bax), while Bcl-2 expression was inhibited [71]. However, even with the well-known and proven antitumor activity of SFN, Rai et al. (2020) found that independent application of SFN contributed to tumor size reduction only when combined with paclitaxel. The authors proposed that SFN could be administered as an adjunct to chemotherapy to reduce side effects [72].

### 5.3. Antitumor Activities of Moringa Isothiocyanates

Although least investigated, MIC has been shown to inhibit cancer growth and promote apoptosis in cancer cells. Notably, MG and one of its acetylated isomers were demonstrated to induce NQO1 enzyme activity as effectively as SFN in hepatocellular carcinoma cells, suggesting that MG acts as an Nrf2 activator as well [73].

Additionally, other MIC-mediated antitumor mechanisms have been recognized as important. Xie et al. (2022) demonstrated that MG induces apoptosis in renal cancer cells both in vitro and in vivo by increasing the Bax/Bcl-2 ratio and inducing cell cycle arrest [74]. In malignant astrocytoma cells, MG stimulated the apoptotic process through p53 and Bax activation and Bcl-2 inhibition. Furthermore, the induction of oxidative stress and subsequent activation of the Nrf2 transcription factor further contributed to tumor cell death [75].

The effects of MG on glioma cells were shown to be time- and dose-dependent, as reported by Xie et al. (2023). They found that MG stimulated tumor cell apoptosis in a manner similar to the previously explained mechanisms without causing harm to normal human gastric mucosal cells [76]. MIC-mediated activation of apoptotic genes, including caspase, p53, Akt/MAPK, and Bax of the proapoptotic Bcl family, has also been observed in human prostate [77], liver [78], and neuroblastoma cancer cells [79].

### 5.4. Anticancer and Cancerogenic Potential of Isothiocyanates

Although current data supports the hypothesis of anti-cancerogenic properties of isothiocyanates, some differences between them were described in the literature. For example, Yuan et al. (2013) compared the antitumor potential of SFN and PEITC in vitro in human glioblastoma cells, as well as in vivo in a murine orthotropic glioblastoma tumor model. While both SFN and PEITC were able to inhibit proliferation and stimulate the apoptosis of three different glioblastoma cell lines, in vivo studies showed that only SFN was able to decrease the tumor weight at a dose of 12.5 mg/kg daily [80]. On the contrary, prostate cancer cells were more sensitive to PEITC than SFN. PEITC was able to inhibit cell replication at a dose of 10 μM while the same effects were seen when prostate cancer cells were treated with 40 μM of SFN [81]. Next, SFN was shown to stimulate apoptosis and suppress the metastasis of bladder cancer cells, both in vitro [82] and in vivo [83] in a dose-dependent manner. While ≥20 μM SFN decreases cell viability and migration of bladder cancer cells, a low concentration of SFN (1–5 μM) promotes their proliferation and migration [84]. Similarly, dual results were seen when bladder cancer was treated with PEITC. The administration of 0.01% to 0.05% of PEITC significantly increased the incidences of papillary or nodular hyperplasia, dysplasia, and transitional cells in rat urinary bladder carcinomas, while doses higher than 0.05% showed antitumor effects [85]. Additionally, when 0.1% PEITC was given to 6-week-old F344 rats for 1, 2, 3, and 7 days, a significant reduction in urinary pH levels compared to the normal control was detected, together with an increase in the thickness of the urinary bladder urothelium and occurrence of inflammation, vacuolation, erosion, and apoptosis/single cell necrosis in the urinary bladder lesion. Histopathological simple hyperplasia was observed when 0.1% PEITC is administered for 14 days, suggesting that the use of PEITC may induce continuous proliferation of bladder epithelial cells, especially in the early stage of bladder carcinogenesis and thus, should be used with caution [81]. When it comes to MIC, current data suggest only positive anticancer effects in bladder cancer [86,87]. However, this may not be a realistic scenario as MIC are the least investigated isothiocyanates and thus, require further investigation to confirm these results. 

Overall, the anti-cancerogenic potential of isothiocyanates might depend on several factors including the type and dose of isothiocyanate used in the treatment, as well as the type of cancer.

## 6. Neuroprotective Properties of Isothiocyanates

### 6.1. Neuroprotective Properties of Phenethyl Isothiocyanate

Phenyl ethyl isothiocyanate has emerged as a promising protective agent in the context of neurodegeneration, showcasing neuroprotective properties through its modulation of various molecular pathways associated with oxidative stress, inflammation, and apoptosis. Experimental models have demonstrated the potential of PEITC to attenuate neurodegenerative processes, presenting a novel avenue for therapeutic intervention [37]. The neuroprotective activity of PEITC is realized through the activation of Nrf2 and its downstream cytoprotective pathways [17]. Despite promising potential, a limited number of studies have explored the use of PEITC in neurodegenerative diseases.

Wang et al. (2019) conducted a study showing that PEITC could promote the repair of injured sensory neurons [88]. However, it is important to note that conflicting findings exist in the literature. One study indicated that PEITC may induce dose-dependent neurotoxic effects in the brain. In an experiment, pregnant adult rats were treated with 15, 60, and 120 mg/kg of PEITC from the 7th to the 16th week of gestation. The study observed impairment of neuro-behavioral development and learning abilities in the offspring exposed to 60 and 120 mg/kg of PEITC, while 15 mg/kg showed no neurotoxic effects [89]. These conflicting results underscore the need for further research to delineate the nuanced effects of PEITC on neurodegenerative processes and to establish safe and effective therapeutic protocols.

### 6.2. Neuroprotective Properties of Sulforaphane

Sulforaphane emerges as the most extensively studied isothiocyanate concerning the prevention of neurodegenerative diseases. Notably, SFN has demonstrated a capacity to counteract amyloid β (Aβ) aggregation in Alzheimer’s disease, with this effect being correlated with the activation of Nrf2 [90]. In cellular studies, Bahn et al. treated SH-SY5Y cells with 1 μM of SFN, resulting in the overexpression of Nrf2 and reduced transcription levels of beta-secretase 1 (BACE1) and BACE1 antisense RNA (BACE1-AS), proteins implicated in amyloidogenic processes [91]. SFN-mediated reduction of oxidative stress and prevention of neural cell damage have shown protective effects in Parkinson’s disease, with the mechanism being Nrf2-ARE-dependent [92]. The observed neuroprotective effects of SFN have been further confirmed in in vivo settings. For instance, Zhang et al. (2015) highlighted the potential therapeutic use of SFN in Alzheimer’s disease. In an Alzheimer’s mouse model, SFN exerted neuroprotective effects by shielding the brain from Aβ-deposits responsible for the degeneration of neurons and synapses. SFN restored endogenous antioxidants in the brain and regulated the activity of glutathione peroxidase (GPX). Additionally, SFN demonstrated a possible anxiolytic effect by influencing reduced locomotor activity in diseased mice [93]. Regular oral administration of SFN at a daily dose of 10 to 50 mg/kg prevented memory impairment characteristic of Alzheimer’s disease and reduced levels of tau, phosphorylated tau, and Aβ-protein—key pathophysiological factors in this disease. SFN achieved these effects by influencing their production and clearance, and increasing the activity of heat shock protein 70 (HSP70) and the *C*-terminus of the HSP70-interacting protein [94]. Furthermore, SFN demonstrated the ability to reduce depressive-like behavior in rats, possibly stemming from memory loss, through effects on serotonin metabolism and transport [95].

In vivo studies by Morroni et al. (2013), Jazwa et al. (2011), and Zhou et al. (2016) confirmed the neuroprotective activity of SFN for the treatment of Parkinson’s disease. SFN exhibited a neuroprotective effect, preventing the degeneration of dopaminergic neurons and significantly improving impaired motor function, coordination, and balance. This effect was attributed to the increased antioxidant potential of the substantia nigra and the inhibition of apoptosis, with SFN enhancing brain antioxidant protection by activating Nrf2 and subsequently the antioxidant enzymes HO-1 and NQO1 [96,97,98].

Yoo et al. (2019) and Li et al. (2013) conducted studies in mice with autoimmune encephalomyelitis to explore the potential of SFN as an adjunctive therapy for multiple sclerosis. SFN demonstrated preventive effects against neuronal degeneration due to its antioxidant and anti-inflammatory properties. The activation of Nrf2 led to increased synthesis and activity of antioxidant enzymes, reducing the number of antigen-specific Th17 cells critical for the development of autoimmune encephalopathy. Additionally, SFN improved the integrity of the blood–brain barrier (BBB) by inhibiting oxidative stress, reducing the expression of matrix metalloproteinase-9 (MMP-9), the tissue inhibitor of metalloproteinase, and consequently protecting levels of the proteins claudin-5 and occludin, which are crucial for maintaining the integrity of the BBB [99,100].

### 6.3. Neuroprotective Properties of Moringa Isothiocyanates

*Moringa* isothiocyanates play a crucial role as antioxidants, effectively neutralizing free radicals and mitigating oxidative stress within the brain. Through the enhancement of the cellular antioxidant defense system, MIC demonstrate a capacity to prevent oxidative damage to neurons, contributing to overall neuroprotection [8]. In parallel to PEITC, MIC exhibit anti-inflammatory effects by suppressing the production of inflammatory cytokines and mediators in the brain [101,102]. Notably, a study conducted by Onasanwo and colleagues observed the potential pharmacological role of *Moringa oleifera* isothiocyanates in preventing the loss of neuronal cells and managing Alzheimer’s disease. They emphasized the critical role of MIC in reducing oxido-inflammatory stress, restoring cholinergic transmission through acetylcholinesterase inhibition, and maintaining neuronal integrity in the brains of mice with exogenously induced neurodegeneration [103].

Furthermore, recent research suggests that treatment with *Moringa* extract improves anxiety-like behavior, hyperactivity, cognitive learning, and memory impairments in mice with developed Alzheimer’s disease. This improvement is attributed to the inhibition of BACE1 and asparagine endopeptidase (AEP), as well as the upregulation of insulin-degrading enzyme (IDE), neprilysin (NEP), and low-density lipoprotein receptor-related protein 1 (LRP1) levels [104]. The same research group previously demonstrated that MIC alleviate hyperphosphorylation and Aβ pathology in a rat model with induced Alzheimer’s disease-like pathology [105]. Additionally, MIC have been shown to promote the expression of neurotrophic factors, such as brain-derived neurotrophic factor (BDNF), which play a crucial role in neuronal survival, growth, and maintenance, thereby contributing to the preservation of cognitive function and preventing neurodegeneration. In a study by Purwoningsih and colleagues, mice treated with *Moringa oleifera* seed oil exhibited amelioration of anxiety-like and depression-like symptoms, as well as memory improvement. This was linked to increased mRNA expression of BDNF, inhibition of acetylcholinesterase (AChE) activity, and prevention of the rise in malondialdehyde levels in the brain [106]. Furthermore, *Moringa oleifera* seed extract shows beneficial effects on congenital aspects. In mice with scopolamine-induced learning and memory impairment, MIC mediated the enhancement of the cholinergic neurotransmission system and neurogenesis through the activation of Akt, ERK1/2, and cAMP response element-binding protein (CREB) signaling pathways, resulting in a reduction of amnesia-related symptoms [107].

Another neuroprotective mechanism of MIC is linked to mitochondrial protection, as demonstrated by Gonzales-Burgos et al. (2021). Their investigation revealed the protective effects of MIC on an H_2_O_2_-induced oxidative stress model in human neuroblastoma cells. In vitro assays indicated that in addition to antioxidative activity involving the reduction of free radicals, a decrease in lipid peroxidation, and enhanced glutathione levels, MIC prevented mitochondrial dysfunction by regulating calcium levels and increasing mitochondrial membrane potential [108].

## 7. Conclusions

Isothiocyanates are inherent natural products that attain bioactivity through the hydrolysis of their precursors, known as glucosinolates. The functional similarity among them arises from the pharmacophore—the isothiocyanate moiety, which interacts effectively with sulfhydryl groups on targeted proteins. A distinguishing characteristic of *Moringa* isothiocyanates lies in their enhanced stability, attributed to an additional sugar group in their structure.

The activation of Nrf2 stands as a pivotal mechanism of action for all isothiocyanates, playing a key role in both antioxidative and anti-inflammatory activities. PEITC, SFN, and MIC collectively exhibit numerous health benefits, ranging from cardio- and nephroprotection to anticancer activity. The small, liposoluble nature of PEITC and SFN enables them to traverse the BBB, facilitating neuroprotective activity. Moreover, current data suggest that, although bigger and less liposoluble, MIC possesses neuroprotective effects, as shown by their ability to reduce oxido-inflammatory stress in neuronal cells. The significant health benefits of isothiocyanates cannot be underestimated and prompt questions on how their potency as antioxidants or anticancer agents varies with their structure. As shown in Table 1, SFN has a simple hydrocarbon backbone with the (−N=C=S) functional group, while PEITC has a phenyl ring. Contrary to others, MIC have a phenyl ring and a rhamnose sugar which confer stability at room temperature [26]. If structural differences influence physical properties, how much influence do they have on function? This question and more are worth exploring as we expand our understanding of the significant health benefits locked in cruciferous vegetables.

## Figures and Tables

**Table 1:** Comparison of the properties of sulforaphane, PEITC, and MIC according to the National Center for Biotechnology Information. PubChem Compound Summary for CID 5350, 16746, and 153557.

Common Name	Sulforaphane	Phenyl Ethyl Isothiocyanates	Moringa Isothiocyanates
Chemical name	1-isothiocyanato-4-methylsulfinylbutane	2-isothiocyanato ethylbenzene	4-[(α-L-rhamnosyloxy)benzyl] isothiocyanate
Chemical Formula	C6H11NOS2	C9H9NS	C16H19NO6S
Chemical Structure	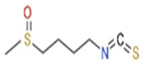	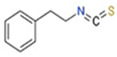	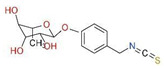
Molecular weight	177.28 g/mol	163.24 g/mol	353.39 g/mol
Appearance	Yellow liquid	Colorless liquid to pale yellow	Solid at room temperature
Solubility	Soluble in lipids	Soluble in lipids	Soluble in lipids
Sources	Broccoli, cabbage, cauliflower, watercress, kale, and Brussels sprouts	Broccoli, cabbage, watercress, garden cress, and Brussels sprouts	*Moringa oleifera* leaves and seeds

## Abbreviations

ARE	Antioxidant response element
GMG	Glucomoringin
HSF1	Heat Shocking Factor 1
GSH	Glutathione S-transferases
GGT	γ Glutamyl Peptidase
MG	Moringin
MIC	Moringa Isothiocyanate
NRF2	Nuclear factor erythroid 2-related factor 2
PEITC	Phenyl ethyl isothiocyanate
GPN	Glucoraphanin
CYP1A1	Cytochrome P450 family 1 subfamily A member 1
CYP3A4	Cytochrome P450 family 3 subfamily A member 4
TNF-α	Tumor necrosis factor α
IL-6	Interleukin 6
TGFB	Transforming growth factor-beta
Bcl-XL	B-cell lymphoma-extra large
Bak	Bcl-2 homologous antagonist killer
CADH1	Cadherin
MAPK/ERK	Mitogen-activated protein kinase/extracellular-signal-regulated kinase signaling
PARP	Poly (ADP-ribose) polymerase
BAX	Bcl-2-associated X protein
BACE1	Beta-secretase 1
BACE1-AS	BACE1 antisense RNA
MMP-9	Matrix metalloproteinase 9
CAT	Catalase
AEP	Asparagine endopeptidase
IDE	Insulin-degrading enzyme
NEP	Neprilysin
LRP1	Low-density lipoprotein receptor-related protein 1
AChE	Acetylcholinesterase
CREB	cAMP response element-binding protein

## References

[1] Verhoeven D.T., Goldbohm R.A., van Poppel G., Verhagen H., van den Brandt P.A. (1996). Epidemiological studies on brassica vegetables and cancer risk. Cancer Epidemiol. Biomark. Prev..

[2] Conaway C.C., Yang Y.M., Chung F.L. (2002). Isothiocyanates as cancer chemopreventive agents: Their biological activities and metabolism in rodents and humans. Curr. Drug Metab..

[3] Bozic D., Baralić K., Živančević K., Miljaković E.A., Ćurčić M., Antonijević B., Djordjević A.B., Bulat Z., Zhang Y., Yang L. (2022). Predicting sulforaphane-induced adverse effects in colon cancer patients via in silico investigation. Biomed. Pharmacother..

[4] Gu H., Mao X., Du M. (2022). Metabolism, absorption, and anti-cancer effects of sulforaphane: An update. Crit. Rev. Food Sci. Nutr..

[5] Hu K., Qi Y., Zhao J., Jiang H., Chen X., Ren J. (2013). Synthesis and biological evaluation of sulforaphane derivatives as potential antitumor agents. Eur. J. Med. Chem..

[6] Iahtisham-Ul-Haq, Khan S., Awan K.A., Iqbal M.J. (2022). Sulforaphane as a potential remedy against cancer: Comprehensive mechanistic review. J. Food Biochem..

[7] Kaiser A.E., Baniasadi M., Giansiracusa D., Giansiracusa M., Garcia M., Fryda Z., Wong T.L., Bishayee A. (2021). Sulforaphane: A Broccoli Bioactive Phytocompound with Cancer Preventive Potential. Cancers.

[8] Kou X., Li B., Olayanju J.B., Drake J.M., Chen N. (2018). Nutraceutical or Pharmacological Potential of *Moringa oleifera* Lam. Nutrients.

[9] Shoaib S., Khan F.B., Alsharif M.A., Malik M.S., Ahmed S.A., Jamous Y.F., Uddin S., Tan C.S., Ardianto C., Tufail S. (2023). Reviewing the Prospective Pharmacological Potential of Isothiocyanates in Fight against Female-Specific Cancers. Cancers.

[10] Mundkar M., Bijalwan A., Soni D., Kumar P. (2022). Neuroprotective potential of *Moringa* oleifera mediated by NF-kB/Nrf2/HO-1 signaling pathway: A review. J. Food Biochem..

[11] Coscueta E.R., Sousa A.S., Reis C.A., Pintado M.M. (2022). Phenylethyl Isothiocyanate: A Bioactive Agent for Gastrointestinal Health. Molecules.

[12] Kamal R.M., Abdull Razis A.F., Mohd Sukri N.S., Perimal E.K., Ahmad H., Patrick R., Djedaini-Pilard F., Mazzon E., Rigaud S. (2022). Beneficial Health Effects of Glucosinolates-Derived Isothiocyanates on Cardiovascular and Neurodegenerative Diseases. Molecules.

[13] Jaafaru M.S., Abd Karim N.A., Enas M.E., Rollin P., Mazzon E., Abdull Razis A.F. (2018). Protective Effect of Glucosinolates Hydrolytic Products in Neurodegenerative Diseases (NDDs). Nutrients.

[14] Aggarwal M., Saxena R., Sinclair E., Fu Y., Jacobs A., Dyba M., Wang X., Cruz I., Berry D., Kallakury B. (2016). Reactivation of mutant p53 by a dietary-related compound phenethyl isothiocyanate inhibits tumor growth. Cell Death Differ..

[15] Smerák P., Polívková Z., Stetina R., Bártová J., Bárta I. (2009). Antimutagenic effect of phenethyl isothiocyanate. Cent. Eur. J. Public Health.

[16] Keum Y.S., Owuor E.D., Kim B.R., Hu R., Kong A.N. (2003). Involvement of Nrf2 and JNK1 in the activation of antioxidant responsive element (ARE) by chemopreventive agent phenethyl isothiocyanate (PEITC). Pharm. Res..

[17] Dayalan Naidu S., Suzuki T., Yamamoto M., Fahey J.W., Dinkova-Kostova A.T. (2018). Phenethyl Isothiocyanate, a Dual Activator of Transcription Factors NRF2 and HSF1. Mol. Nutr. Food Res..

[18] Nandini D.B., Rao R.S., Deepak B.S., Reddy P.B. (2020). Sulforaphane in broccoli: The green chemoprevention!! Role in cancer prevention and therapy. J. Oral Maxillofac. Pathol..

[19] Liang J., Hänsch G.M., Hübner K., Samstag Y. (2019). Sulforaphane as anticancer agent: A double-edged sword? Tricky balance between effects on tumor cells and immune cells. Adv. Biol. Regul..

[20] Rafiei H., Ashrafizadeh M., Ahmadi Z. (2020). MicroRNAs as novel targets of sulforaphane in cancer therapy: The beginning of a new tale?. Phytother. Res..

[21] Sestili P., Fimognari C. (2015). Cytotoxic and Antitumor Activity of Sulforaphane: The Role of Reactive Oxygen Species. Biomed. Res. Int..

[22] Kamal M.M., Akter S., Lin C.-N., Nazzal S. (2020). Sulforaphane as an anticancer molecule: Mechanisms of action, synergistic effects, enhancement of drug safety, and delivery systems. Arch. Pharm. Res..

[23] Mangla B., Javed S., Sultan M.H., Kumar P., Kohli K., Najmi A., Alhazmi H.A., Al Bratty M., Ahsan W. (2021). Sulforaphane: A review of its therapeutic potentials, advances in its nanodelivery, recent patents, and clinical trials. Phyther. Res..

[24] Houghton C.A., Fassett R.G., Coombes J.S. (2016). Sulforaphane and Other Nutrigenomic Nrf2 Activators: Can the Clinician’s Expectation Be Matched by the Reality?. Oxid. Med. Cell. Longev..

[25] Fahey J.W., Wade K.L., Stephenson K.K., Shi Y., Liu H., Panjwani A.A., Warrick C.R., Olson M.E. (2019). A Strategy to Deliver Precise Oral Doses of the Glucosinolates or Isothiocyanates from *Moringa oleifera* Leaves for Use in Clinical Studies. Nutrients.

[26] Waterman C., Cheng D.M., Rojas-Silva P., Poulev A., Dreifus J., Lila M.A., Raskin I. (2014). Stable, water extractable isothiocyanates from *Moringa* oleifera leaves attenuate inflammation in vitro. Phytochemistry.

[27] Borgonovo G., De Petrocellis L., Schiano Moriello A., Bertoli S., Leone A., Battezzati A., Mazzini S., Bassoli A. (2020). Moringin, A Stable Isothiocyanate from *Moringa oleifera*, Activates the Somatosensory and Pain Receptor TRPA1 Channel In Vitro. Molecules.

[28] Wu X., Zhou Q.H., Xu K. (2009). Are isothiocyanates potential anti-cancer drugs?. Acta Pharmacol. Sin..

[29] Esteve M. (2020). Mechanisms Underlying Biological Effects of Cruciferous Glucosinolate-Derived Isothiocyanates/Indoles: A Focus on Metabolic Syndrome. Front. Nutr..

[30] Ioannides C., Konsue N. (2015). A principal mechanism for the cancer chemopreventive activity of phenethyl isothiocyanate is modulation of carcinogen metabolism. Drug Metab. Rev..

[31] Morris M.E., Dave R.A. (2014). Pharmacokinetics and pharmacodynamics of phenethyl isothiocyanate: Implications in breast cancer prevention. AAPS J..

[32] Kaschula C.H., Hunter R., Atta-ur-Rahman (2016). Chapter 1—Synthesis and Structure–Activity Relations in Allylsulfide and Isothiocyanate Compounds from Garlic and Broccoli Against In Vitro Cancer Cell Growth.

[33] Wolff K., Jaja-Chimedza A., Kim Y., Waterman C., Poulev A., Raskin I., Ribnicky D. (2020). Moringa isothiocyanate-1 is bioaccessible and bioavailable as a stable unmodified compound. Phytochem. Lett..

[34] Wang F., Bao Y., Zhang C., Zhan L., Khan W., Siddiqua S., Ahmad S., Capanoglu E., Skalicka-Woźniak K., Zou L. (2022). Bioactive components and anti-diabetic properties of Moringa oleifera Lam. Crit. Rev. Food Sci. Nutr..

[35] Wu Y.Y., Xu Y.M., Lau A.T.Y. (2021). Anti-Cancer and Medicinal Potentials of *Moringa* Isothiocyanate. Molecules.

[36] Gupta P., Adkins C., Lockman P., Srivastava S.K. (2013). Metastasis of Breast Tumor Cells to Brain Is Suppressed by Phenethyl Isothiocyanate in a Novel In Vivo Metastasis Model. PLoS ONE.

[37] Giacoppo S., Galuppo M., Montaut S., Iori R., Rollin P., Bramanti P., Mazzon E. (2015). An overview on neuroprotective effects of isothiocyanates for the treatment of neurodegenerative diseases. Fitoterapia.

[38] Saha S., Buttari B., Panieri E., Profumo E., Saso L. (2020). An Overview of Nrf2 Signaling Pathway and Its Role in Inflammation. Molecules.

[39] Ahmed S.M., Luo L., Namani A., Wang X.J., Tang X. (2017). Nrf2 signaling pathway: Pivotal roles in inflammation. Biochim. Biophys. Acta Mol. Basis Dis..

[40] Ernst I.M., Wagner A.E., Schuemann C., Storm N., Höppner W., Döring F., Stocker A., Rimbach G. (2011). Allyl-, butyl- and phenylethyl-isothiocyanate activate Nrf2 in cultured fibroblasts. Pharmacol. Res..

[41] Keum Y.S. (2011). Regulation of the Keap1/Nrf2 system by chemopreventive sulforaphane: Implications of posttranslational modifications. Ann. N. Y. Acad. Sci..

[42] Ahn Y.H., Hwang Y., Liu H., Wang X.J., Zhang Y., Stephenson K.K., Boronina T.N., Cole R.N., Dinkova-Kostova A.T., Talalay P. (2010). Electrophilic tuning of the chemoprotective natural product sulforaphane. Proc. Natl. Acad. Sci. USA.

[43] Eisa N.H., Khodir A.E., El-Sherbiny M., Elsherbiny N.M., Said E. (2021). Phenethyl isothiocyanate attenuates diabetic nephropathy via modulation of glycative/oxidative/inflammatory signaling in diabetic rats. Biomed. Pharmacother..

[44] Gwon M.H., Yun J.M. (2021). Phenethyl Isothiocyanate Improves Lipid Metabolism and Inflammation via *mTOR/PPARγ/AMPK* Signaling in the Adipose Tissue of Obese Mice. J. Med. Food.

[45] Park H.J., Kim S.J., Park S.J., Eom S.H., Gu G.J., Kim S.H., Youn H.S. (2013). Phenethyl isothiocyanate regulates inflammation through suppression of the TRIF-dependent signaling pathway of Toll-like receptors. Life Sci..

[46] Pan J., Wang R., Pei Y., Wang D., Wu N., Ji Y., Tang Q., Liu L., Cheng K., Liu Q. (2023). Sulforaphane alleviated vascular remodeling in hypoxic pulmonary hypertension via inhibiting inflammation and oxidative stress. J. Nutr. Biochem..

[47] Egbujor M.C., Petrosino M., Zuhra K., Saso L. (2022). The Role of Organosulfur Compounds as Nrf2 Activators and Their Antioxidant Effects. Antioxidants.

[48] Gu J., Cheng Y., Wu H., Kong L., Wang S., Xu Z., Zhang Z., Tan Y., Keller B.B., Zhou H. (2017). Metallothionein Is Downstream of Nrf2 and Partially Mediates Sulforaphane Prevention of Diabetic Cardiomyopathy. Diabetes.

[49] Sun Y., Zhou S., Guo H., Zhang J., Ma T., Zheng Y., Zhang Z., Cai L. (2020). Protective effects of sulforaphane on type 2 diabetes-induced cardiomyopathy via AMPK-mediated activation of lipid metabolic pathways and NRF2 function. Metabolism.

[50] Cheng D., Gao L., Su S., Sargsyan D., Wu R., Raskin I., Kong A.N. (2019). *Moringa* Isothiocyanate Activates Nrf2: Potential Role in Diabetic Nephropathy. AAPS J..

[51] Sailaja B.S., Aita R., Maledatu S., Ribnicky D., Verzi M.P., Raskin I. (2021). *Moringa* isothiocyanate-1 regulates Nrf2 and NF-κB pathway in response to LPS-driven sepsis and inflammation. PLoS ONE.

[52] Chen L., Fan D., Guo F., Deng J., Fu L. (2023). The Effect of *Moringa* Isothiocyanate-1 on Renal Damage in Diabetic Nephropathy. Iran. J. Kidney Dis..

[53] Shoaib S., Tufail S., Sherwani M.A., Yusuf N., Islam N. (2021). Phenethyl Isothiocyanate Induces Apoptosis Through ROS Generation and Caspase-3 Activation in Cervical Cancer Cells. Front. Pharmacol..

[54] Lai K.C., Chueh F.S., Ma Y.S., Chou Y.C., Chen J.C., Liao C.L., Huang Y.P., Peng S.F. (2023). Phenethyl isothiocyanate and irinotecan synergistically induce cell apoptosis in colon cancer HCT 116 cells in vitro. Environ. Toxicol..

[55] Lv H., Zhen C., Liu J., Shang P. (2020). *β*-Phenethyl Isothiocyanate Induces Cell Death in Human Osteosarcoma through Altering Iron Metabolism, Disturbing the Redox Balance, and Activating the MAPK Signaling Pathway. Oxid. Med. Cell. Longev..

[56] Wu R., Li S., Sargsyan D., Yin R., Kuo H.C., Peter R., Wang L., Hudlikar R., Liu X., Kong A.N. (2021). DNA methylome, transcriptome, and prostate cancer prevention by phenethyl isothiocyanate in TRAMP mice. Mol. Carcinog..

[57] Stan S.D., Singh S.V., Whitcomb D.C., Brand R.E. (2014). Phenethyl isothiocyanate inhibits proliferation and induces apoptosis in pancreatic cancer cells in vitro and in a MIAPaca2 xenograft animal model. Nutr. Cancer.

[58] Zhang T., Zhang W., Hao M. (2021). Phenethyl isothiocyanate reduces breast cancer stem cell-like properties by epigenetic reactivation of CDH1. Oncol. Rep..

[59] Shin J.M., Lim E., Cho Y.S., Nho C.W. (2021). Cancer-preventive effect of phenethyl isothiocyanate through tumor microenvironment regulation in a colorectal cancer stem cell xenograft model. Phytomedicine.

[60] Krajka-Kuźniak V., Cykowiak M., Szaefer H., Kleszcz R., Baer-Dubowska W. (2020). Combination of xanthohumol and phenethyl isothiocyanate inhibits NF-κB and activates Nrf2 in pancreatic cancer cells. Toxicol. In Vitro.

[61] Kasukabe T., Honma Y., Okabe-Kado J., Higuchi Y., Kato N., Kumakura S. (2016). Combined treatment with cotylenin A and phenethyl isothiocyanate induces strong antitumor activity mainly through the induction of ferroptotic cell death in human pancreatic cancer cells. Oncol. Rep..

[62] Li Q., Zhan M., Chen W., Zhao B., Yang K., Yang J., Yi J., Huang Q., Mohan M., Hou Z. (2016). Phenylethyl isothiocyanate reverses cisplatin resistance in biliary tract cancer cells via glutathionylation-dependent degradation of Mcl-1. Oncotarget.

[63] Castro N.P., Rangel M.C., Merchant A.S., MacKinnon G., Cuttitta F., Salomon D.S., Kim Y.S. (2019). Sulforaphane Suppresses the Growth of Triple-negative Breast Cancer Stem-like Cells In vitro and In vivo. Cancer Prev. Res..

[64] Kanematsu S., Yoshizawa K., Uehara N., Miki H., Sasaki T., Kuro M., Lai Y.C., Kimura A., Yuri T., Tsubura A. (2011). Sulforaphane inhibits the growth of KPL-1 human breast cancer cells in vitro and suppresses the growth and metastasis of orthotopically transplanted KPL-1 cells in female athymic mice. Oncol. Rep..

[65] Chen X., Jiang Z., Zhou C., Chen K., Li X., Wang Z., Wu Z., Ma J., Ma Q., Duan W. (2018). Activation of Nrf2 by Sulforaphane Inhibits High Glucose-Induced Progression of Pancreatic Cancer via AMPK Dependent Signaling. Cell. Physiol. Biochem..

[66] Chen Y., Chen J.Q., Ge M.M., Zhang Q., Wang X.Q., Zhu J.Y., Xie C.F., Li X.T., Zhong C.Y., Han H.Y. (2019). Sulforaphane inhibits epithelial-mesenchymal transition by activating extracellular signal-regulated kinase 5 in lung cancer cells. J. Nutr. Biochem..

[67] Li X., He S., Ma B. (2020). Autophagy and autophagy-related proteins in cancer. Mol. Cancer.

[68] Lu Z., Ren Y., Yang L., Jia A., Hu Y., Zhao Y., Zhao W., Yu B., Zhao W., Zhang J. (2021). Inhibiting autophagy enhances sulforaphane-induced apoptosis via targeting NRF2 in esophageal squamous cell carcinoma. Acta Pharm. Sin. B.

[69] Byun S., Shin S.H., Park J., Lim S., Lee E., Lee C., Sung D., Farrand L., Lee S.R., Kim K.H. (2016). Sulforaphene suppresses growth of colon cancer-derived tumors via induction of glutathione depletion and microtubule depolymerization. Mol. Nutr. Food Res..

[70] Xu Y., Han X., Li Y., Min H., Zhao X., Zhang Y., Qi Y., Shi J., Qi S., Bao Y. (2019). Sulforaphane Mediates Glutathione Depletion via Polymeric Nanoparticles to Restore Cisplatin Chemosensitivity. ACS Nano.

[71] Lin L.C., Yeh C.T., Kuo C.C., Lee C.M., Yen G.C., Wang L.S., Wu C.H., Yang W.C., Wu A.T. (2012). Sulforaphane potentiates the efficacy of imatinib against chronic leukemia cancer stem cells through enhanced abrogation of Wnt/β-catenin function. J. Agric. Food Chem..

[72] Rai R., Gong Essel K., Mangiaracina Benbrook D., Garland J., Daniel Zhao Y., Chandra V. (2020). Preclinical Efficacy and Involvement of AKT, mTOR, and ERK Kinases in the Mechanism of Sulforaphane against Endometrial Cancer. Cancers.

[73] Jaja-Chimedza A., Graf B.L., Simmler C., Kim Y., Kuhn P., Pauli G.F., Raskin I. (2017). Biochemical characterization and anti-inflammatory properties of an isothiocyanate-enriched *Moringa* (*Moringa* oleifera) seed extract. PLoS ONE.

[74] Xie J., Qian Y.Y., Yang Y., Peng L.J., Mao J.Y., Yang M.R., Tian Y., Sheng J. (2022). Isothiocyanate from *Moringa oleifera* Seeds Inhibits the Growth and Migration of Renal Cancer Cells by Regulating the PTP1B-dependent Src/Ras/Raf/ERK Signaling Pathway. Front. Cell Dev. Biol..

[75] Rajan T.S., De Nicola G.R., Iori R., Rollin P., Bramanti P., Mazzon E. (2016). Anticancer activity of glucomoringin isothiocyanate in human malignant astrocytoma cells. Fitoterapia.

[76] Xie J., Yang M.R., Hu X., Hong Z.S., Bai Y.Y., Sheng J., Tian Y., Shi C.Y. (2023). *Moringa oleifera* Lam. Isothiocyanate Quinazolinone Derivatives Inhibit U251 Glioma Cell Proliferation through Cell Cycle Regulation and Apoptosis Induction. Int. J. Mol. Sci..

[77] Abd Karim N.A., Adam A.H.B., Jaafaru M.S., Rukayadi Y., Abdull Razis A.F. (2023). Apoptotic Potential of Glucomoringin Isothiocyanate (GMG-ITC) Isolated from *Moringa oleifera* Lam Seeds on Human Prostate Cancer Cells (PC-3). Molecules.

[78] Antonini E., Iori R., Ninfali P., Scarpa E.S. (2018). A Combination of Moringin and Avenanthramide 2f Inhibits the Proliferation of Hep3B Liver Cancer Cells Inducing Intrinsic and Extrinsic Apoptosis. Nutr. Cancer.

[79] Cirmi S., Ferlazzo N., Gugliandolo A., Musumeci L., Mazzon E., Bramanti A., Navarra M. (2019). Moringin from *Moringa oleifera* Seeds Inhibits Growth, Arrests Cell-Cycle, and Induces Apoptosis of SH-SY5Y Human Neuroblastoma Cells through the Modulation of NF-κB and Apoptotic Related Factors. Int. J. Mol. Sci..

[80] Yuan L., Ren X., Jaboin J., McConathy J., Rich K.M. (2013). Isothiocyanates promote cell death and sensitize glioblastoma cells to radiation. Cancer Res..

[81] Hać A., Brokowska J., Rintz E., Bartkowski M., Węgrzyn G., Herman-Antosiewicz A. (2020). Mechanism of selective anticancer activity of isothiocyanates relies on differences in DNA damage repair between cancer and healthy cells. Eur. J. Nutr..

[82] Wang F., Liu P., An H., Zhang Y. (2020). Sulforaphane suppresses the viability and metastasis, and promotes the apoptosis of bladder cancer cells by inhibiting the expression of FAT-1. Int. J. Mol. Med..

[83] Huang L., He C., Zheng S., Wu C., Ren M., Shan Y. (2022). AKT1/HK2 Axis-mediated Glucose Metabolism: A Novel Therapeutic Target of Sulforaphane in Bladder Cancer. Mol. Nutr. Food Res..

[84] Mastuo T., Miyata Y., Yuno T., Mukae Y., Otsubo A., Mitsunari K., Ohba K., Sakai H. (2020). Molecular Mechanisms of the Anti-Cancer Effects of Isothiocyanates from Cruciferous Vegetables in Bladder Cancer. Molecules.

[85] Ogawa K., Hirose M., Sugiura S., Cui L., Imaida K., Ogiso T., Shirai T. (2001). Dose-dependent promotion by phenylethyl isothiocyanate, a known chemopreventer, of two-stage rat urinary bladder and liver carcinogenesis. Nutr. Cancer.

[86] Taha N.R., Amin H.A., Sultan A.A. (2015). The protective effect of Moringa oleifera leaves against cyclophosphamide-induced urinary bladder toxicity in rats. Tissue Cell.

[87] Sreelatha S., Jeyachitra A., Padma P.R. (2011). Antiproliferation and induction of apoptosis by Moringa oleifera leaf extract on human cancer cells. Food Chem. Toxicol..

[88] Wang Z., Yuan W., Li B., Chen X., Zhang Y., Chen C., Yu M., Xiu Y., Li W., Cao J. (2019). PEITC promotes neurite growth in primary sensory neurons via the miR-17-5p/STAT3/GAP-43 axis. J. Drug Target..

[89] Zhi Y., Liu H., Geng G., Yu Z., Xu H. (2011). Changes on neurobehavioral development of the offspring in rats prenataly exposed by phenethyl isothiocyanate. Wei Sheng Yan Jiu.

[90] Hou T.T., Yang H.Y., Wang W., Wu Q.Q., Tian Y.R., Jia J.P. (2018). Sulforaphane Inhibits the Generation of Amyloid-β Oligomer and Promotes Spatial Learning and Memory in Alzheimer’s Disease (PS1V97L) Transgenic Mice. J. Alzheimers Dis..

[91] Bahn G., Park J.-S., Yun U.J., Lee Y.J., Choi Y., Park J.S., Baek S.H., Choi B.Y., Cho Y.S., Kim H.K. (2019). NRF2/ARE pathway negatively regulates BACE1 expression and ameliorates cognitive deficits in mouse Alzheimer’s models. Proc. Natl. Acad. Sci. USA.

[92] Bao B., Zhang M.Q., Chen Z.Y., Wu X.B., Xia Z.B., Chai J.Y., Yin X.P. (2019). Sulforaphane prevents PC12 cells from oxidative damage via the Nrf2 pathway. Mol. Med. Rep..

[93] Zhang R., Miao Q.W., Zhu C.X., Zhao Y., Liu L., Yang J., An L. (2015). Sulforaphane ameliorates neurobehavioral deficits and protects the brain from amyloid β deposits and peroxidation in mice with Alzheimer-like lesions. Am. J. Alzheimers Dis. Other Dement..

[94] Lee S., Choi B.R., Kim J., LaFerla F.M., Park J.H.Y., Han J.S., Lee K.W., Kim J. (2018). Sulforaphane Upregulates the Heat Shock Protein Co-Chaperone CHIP and Clears Amyloid-β and Tau in a Mouse Model of Alzheimer’s Disease. Mol. Nutr. Food Res..

[95] Wang W., Wei C., Quan M., Li T., Jia J. (2020). Sulforaphane Reverses the Amyloid-β Oligomers Induced Depressive-Like Behavior. J. Alzheimers Dis..

[96] Morroni F., Tarozzi A., Sita G., Bolondi C., Zolezzi Moraga J.M., Cantelli-Forti G., Hrelia P. (2013). Neuroprotective effect of sulforaphane in 6-hydroxydopamine-lesioned mouse model of Parkinson’s disease. Neurotoxicology.

[97] Jazwa A., Rojo A.I., Innamorato N.G., Hesse M., Fernández-Ruiz J., Cuadrado A. (2011). Pharmacological targeting of the transcription factor Nrf2 at the basal ganglia provides disease modifying therapy for experimental parkinsonism. Antioxid. Redox Signal..

[98] Zhou Q., Chen B., Wang X., Wu L., Yang Y., Cheng X., Hu Z., Cai X., Yang J., Sun X. (2016). Sulforaphane protects against rotenone-induced neurotoxicity in vivo: Involvement of the mTOR, Nrf2, and autophagy pathways. Sci. Rep..

[99] Yoo I.H., Kim M.J., Kim J., Sung J.J., Park S.T., Ahn S.W. (2019). The Anti-Inflammatory Effect of Sulforaphane in Mice with Experimental Autoimmune Encephalomyelitis. J. Korean Med. Sci..

[100] Li B., Cui W., Liu J., Li R., Liu Q., Xie X.H., Ge X.L., Zhang J., Song X.J., Wang Y. (2013). Sulforaphane ameliorates the development of experimental autoimmune encephalomyelitis by antagonizing oxidative stress and Th17-related inflammation in mice. Exp. Neurol..

[101] Ghimire S., Subedi L., Acharya N., Gaire B.P. (2021). *Moringa oleifera*: A Tree of Life as a Promising Medicinal Plant for Neurodegenerative Diseases. J. Agric. Food Chem..

[102] Azlan U.K., Khairul Annuar N.A., Mediani A., Aizat W.M., Damanhuri H.A., Tong X., Yanagisawa D., Tooyama I., Wan Ngah W.Z., Jantan I. (2023). An insight into the neuroprotective and anti-neuroinflammatory effects and mechanisms of *Moringa oleifera*. Front. Pharmacol..

[103] Onasanwo S.A., Adamaigbo V.O., Adebayo O.G., Eleazer S.E. (2021). *Moringa* oleifera-supplemented diet protect against cortico-hippocampal neuronal degeneration in scopolamine-induced spatial memory deficit in mice: Role of oxido-inflammatory and cholinergic neurotransmission pathway. Metab. Brain Dis..

[104] Mahaman Y.A.R., Feng J., Huang F., Salissou M.T.M., Wang J., Liu R., Zhang B., Li H., Zhu F., Wang X. (2022). *Moringa oleifera* Alleviates Aβ Burden and Improves Synaptic Plasticity and Cognitive Impairments in APP/PS1 Mice. Nutrients.

[105] Mahaman Y.A.R., Huang F., Wu M., Wang Y., Wei Z., Bao J., Salissou M.T.M., Ke D., Wang Q., Liu R. (2018). *Moringa oleifera* Alleviates Homocysteine-Induced Alzheimer’s Disease-Like Pathology and Cognitive Impairments. J. Alzheimers Dis..

[106] Purwoningsih E., Arozal W., Lee H.J., Barinda A.J., Sani Y., Munim A. (2022). The Oil Formulation Derived from *Moringa oleifera* Seeds Ameliorates Behavioral Abnormalities in Water-immersion Restraint Stress Mouse Model. J. Exp. Pharmacol..

[107] Zhou J., Yang W.S., Suo D.Q., Li Y., Peng L., Xu L.X., Zeng K.Y., Ren T., Wang Y., Zhou Y. (2018). *Moringa oleifera* Seed Extract Alleviates Scopolamine-Induced Learning and Memory Impairment in Mice. Front. Pharmacol..

[108] González-Burgos E., Ureña-Vacas I., Sánchez M., Gómez-Serranillos M.P. (2021). Nutritional Value of *Moringa oleifera* Lam. Leaf Powder Extracts and Their Neuroprotective Effects via Antioxidative and Mitochondrial Regulation. Nutrients.

